# Soft Neurological Signs in Childhood by Measurement of Arm Movements Using Acceleration and Angular Velocity Sensors

**DOI:** 10.3390/s151025793

**Published:** 2015-10-12

**Authors:** Miki Kaneko, Yushiro Yamashita, Osamu Inomoto, Keiji Iramina

**Affiliations:** 1 Graduate School of Systems Life Sciences, Kyushu University, 3-1-1 Maidashi, Higashi-Ku, Fukuoka-Shi, Fukuoka 812-8582, Japan; E-Mail: iramina@inf.kyushu-u.ac.jp; 2 Department of Pediatrics and Child Health, Kurume University School of Medicine, 67 Asahi-Machi, Kurume-Shi, Fukuoka 830-0011, Japan; E-Mail: yushiro@med.kurume-u.ac.jp; 3 Graduate School of Education, Hyogo University of Teacher Education, 942-1 Shimokume, Kato-Shi, Hyogo 673-1494, Japan; E-Mail: inomoto@hyogo-u.ac.jp; 4 Faculty of Information Science and Electrical Engineering, Kyushu University, 744 Motooka, Nishi-Ku, Fukuoka-Shi, Fukuoka 819-0395, Japan

**Keywords:** acceleration and angular velocity sensors, motion analysis, soft neurological signs, pronation, supination, typically developing children

## Abstract

Soft neurological signs (SNS) are evident in the motor performance of children and disappear as the child grows up. Therefore SNS are used as criteria for evaluating age-appropriate development of neurological function. The aim of this study was to quantify SNS during arm movement in childhood. In this study, we focused on pronation and supination, which are arm movements included in the SNS examination. Two hundred and twenty-three typically developing children aged 4–12 years (107 boys, 116 girls) and 18 adults aged 21–26 years (16 males, two females) participated in the experiment. To quantify SNS during pronation and supination, we calculated several evaluation index scores: bimanual symmetry, compliance, postural stability, motor speed and mirror movement. These index scores were evaluated using data obtained from sensors attached to the participants’ hands and elbows. Each score increased as age increased. Results obtained using our system showed developmental changes that were consistent with criteria for SNS. We were able to successfully quantify SNS during pronation and supination. These results indicate that it may be possible to use our system as quantitative criteria for evaluating development of neurological function.

## 1. Introduction

Soft neurological signs (SNS) are minor neurological findings and one of the variables examined in a pediatric neurological examination [1,2]. SNS are likely to appear in the motor performance of typically developing young children and disappear as the child grows up [1]. Therefore SNS play an important role as criteria for evaluating age-appropriate development in neurological function. There are various motor tasks that are used to evaluate SNS, such as the finger-to-nose test, finger opposition test, visual pursuit movements and heel-to-toe walking. Current conventional methods to evaluate SNS are based on visual observations by doctors when evaluating whether a child’s development stage of brain function is age-appropriate. Several studies have reported a relation between developmental changes in brain or neurological function of childhood and SNS identified by visual observation [2,3,4,5,6,7]. There has also been a longitudinal study of SNS [8]. These previous studies have reported that, in typically developing (TD) children, SNS gradually fade away as children grow older [9,10,11,12]. However, visual observation is easily influenced by various biases. Therefore, establishment of criteria for more quantitative evaluation is desirable.

The aim of this study was to quantify age-appropriate developmental changes of SNS in childhood. We focused on pronation and supination of the forearms, which is one motor task is used to evaluate SNS. Pronation and supination of the forearms involves bending the elbows to 90 degrees, and then rotating the palm and the back of the hands. This motor task is used to evaluate SNS in children aged 4 years and above. Examples of evaluation indices of pronation and supination are the rotational speed of forearms, the postural stability of the elbows, and the presence or absence of mirror movements [13]. In pronation and supination, mirror movements are involuntary movement of one hand that can be simultaneously occurred when the contralateral hand voluntary moved by the subject [14].

Previous studies have quantitatively measured pronation and supination. Examples of systems that have been used for this purpose are a three-dimensional motion analyzer [15], a microcomputer-based device [16] and an ultrasound-based recording device [17]. However, these methods are restricted to a particular location or require the use of large-scale mechanical equipment. There is little information about age-appropriate developmental changes in pronation and supination throughout childhood that has been obtained using quantitative evaluation methods. An evaluation system that is more portable and can be easily used anywhere is required for measuring pronation and supination in children.

We have used wireless acceleration and angular velocity sensors as a more simple method to detect and quantify age-appropriate developmental change of SNS in pronation and supination [18]. The sensors were attached to the limbs of the participants, with one sensor on each hand and one sensor on each elbow, to enable evaluation of all conventional indices of pronation and supination. A total of 223 TD children aged 4–12 years old (107 boys, 116 girls) participated in the experiment. We proposed indices to evaluate characteristics of pronation and supination and compared indices across age groups to establish quantitative age-appropriate criteria for pronation and supination.

## 2. Experimental Design

### 2.1. System Configuration

**Figure 1:**
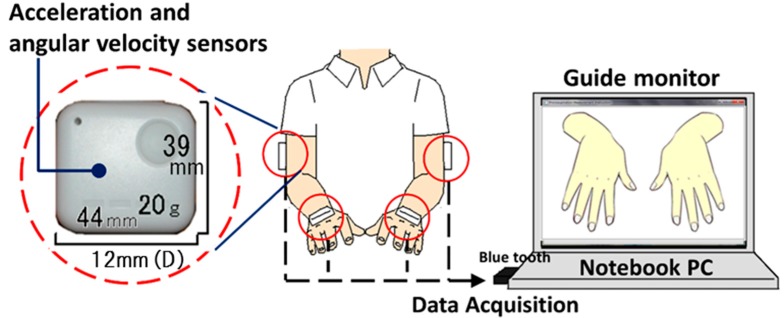
System used to evaluate pronation and supination of the forearms. For the imitative motor task, the participant imitated the motions shown on the guide monitor. For the maximal-effort motor task, the participant moved their hands as fast as possible.

**Figure 2:**
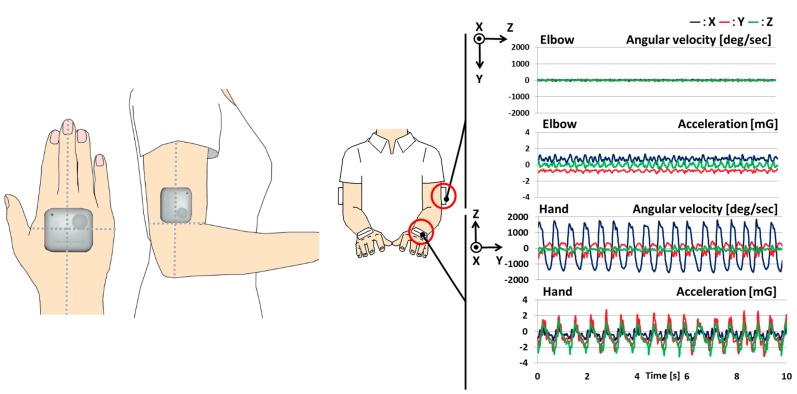
The position of sensors and example waveforms from the sensors in each of the three axes during pronation and supination of the forearm.

As shown in Figure 1, the system comprised four wearable sensors (WAA-006, WAA-010, ATR-Promotions, Kyoto, Japan), a guide monitor (CLAiR SK-DTV 133JW2, Sknet, Kanagawa, Japan) and a notebook PC (VAIO VGN-NW91FS, Sony, Tokyo, Japan). Signals were transmitted by Bluetooth to the notebook PC. The sensors comprised three-axis acceleration and three-axis angular velocity sensors. The sensors were attached to the limbs of the subject, with one sensor on each hand and one sensor on each elbow, as shown in Figure 2. The coordinate system of the sensors is illustrated in Figure 2.

### 2.2. Procedure

Before starting the experiment, all participants received an explanation of the motor tasks. Participants bent their elbows to 90° and were instructed to maintain this basic posture throughout the tasks. Four type of task were performed: an imitative motor task and a maximal-effort motor task performed with both hands, with only the dominant hand, and with only the non-dominant hand.

For the imitative motor task, the participant stood in front of the guide monitor and imitated the motions of the demonstration guide. The demonstration guide showed a motion in which both hands were pronated and supinated 80 times per minute.

For the maximal-effort motor task, the participant pronated and supinated the hand as fast as possible. This task was performed with both hands, with only the dominant hand and with only the non-dominant hand. For the single-hand movements, the participants were instructed to maintain the basic posture with the other hand (non-rotating hand). Each task was performed for 10 s.

### 2.3. Participants

Two hundred twenty-three TD children aged 4–12 years (107 boys, 116 girls) participated in the experiment (Table 1). TD children were recruited from Fukuoka Municipal Elementary School and a kindergarten associated with the Hyogo University of Teacher Education. These children had never previously participated in our experiment. We also measured 18 participants aged 21–26 years old (16 males, two females) to define ideal motor function in pronation and supination. Before starting the experiment, all participants and their parents received an explanation of the aim of this study, and of the procedures and hazards of the experiment. All participants agreed to participate. The study was approved by the Kyushu University Ethics Committee.

**Table 1:** Number of study participants.

Age (years)	Male	Female	Total
4	6	3	9
5	9	5	14
6	4	7	11
7	13	19	32
8	22	14	36
9	19	21	40
10	17	20	37
11	8	18	26
12	9	9	18
21–26	16	2	18
Total	123	118	241

## 3. Analysis Design

### 3.1. Evaluation Indices

We quantified the following conventional evaluation indices of pronation and supination: postural stability, rotation speed and mirror movement. We also quantified the following new indices of pronation and supination: bimanual symmetry and compliance. These five indices were quantified to evaluate various characteristics of pronation and supination.

Bimanual symmetry is an index of the precision of symmetrical movement between the left hand and the right hand during an imitative motor task. Compliance is an index of the correlation between the participants’ motion speed and motion speed of the demonstration guide. Postural stability of the hands is an index reflecting the up-and-down movement of a subject’s hands. Postural stability of the elbows is an index reflecting whether the subject’s elbows move away from their sides. Mirror movement is an index reflecting involuntary movement of one hand that can be simultaneously occurred when the contralateral hand voluntary moved by the subject.

The waveforms of the three-axis gyroscope and a three-axis accelerometer, along with the measurement coordinate system of pronation and supination, are illustrated in Figure 2. The sampling frequency was 100 Hz. A 6-Hz low-pass filter was applied. Forearm movement data obtained from sensors placed on the dorsal aspects of both hands and elbows of participants were used to quantify the indices of movement.

#### Analyzed Parameters

We calculated two parameters of bimanual symmetry: the correlation coefficient of acceleration along the Z axis between the right hand and the left hand, and time delay of acceleration in the Z axis between the right hand and left hand. If the phase between the right hand waveform and the left hand waveform increases, time delay increases. In the imitative motor task, compliance was calculated using the difference between the rotational frequency of the demonstration guide (1.33 Hz) and the peak frequency of the continuous fast Fourier transform (FFT) of the acceleration waveform along the Z axis of the participant’s motion. Postural stability of the hands and elbows was calculated using the absolute value of the total sum of acceleration along the X axis and Z axis. If participants moved their arms and elbows while pronating and supinating their forearms, this parameter increased. Rotational speed was calculated using the peak frequency of the continuous FFT of angular velocity in the X axis. In the maximum effort task of one hand, postural stability of the hands was calculated using the peak frequency of the continuous FFT of angular velocity in the Y axis and Z axis. Postural stability of the elbows was calculated using the absolute value of the total sum of acceleration along the X axis. In both hands of postural stability, we used the absolute value of the total sum and the peak frequency of the continuous FFT of acceleration along the X axis and the Z axis. Mirror movements were calculated using the absolute value of the total sum of acceleration along the Z axis and the peak frequency of the continuous FFT of angular velocity along the X axis.

### 3.2. Evaluation Score and Statistics

We used the mean value and standard deviation of parameters in participants aged 21–26 years as reference data for normalizing parameters in all participants using the following formula:

$$y_{h}=80+\frac{10}{{\text{σ}}_{a}}(-x_{h}+{\text{μ}}_{a})$$

This formula refers to the formula for calculating T-scores. *x_h_* is the value of the parameter in TD children. μ*_a_* and σ*_a_* are the mean value and standard deviation of the parameter in participants aged 21–26 years old. As shown in the formula, we set the mean value of participants aged 21–26 years old (*i.e.*, the ideal score) to 80 points and the score range for all participants to 0–100 points. The *y_h_* scores of TD children indicate how far they are from the ideal score. We used Tukey’s Honest Significant Difference test to evaluate age-related differences in pronation and supination. This test can be used when there are a different number of samples in each group.

## 4. Results

**Figure 3:**
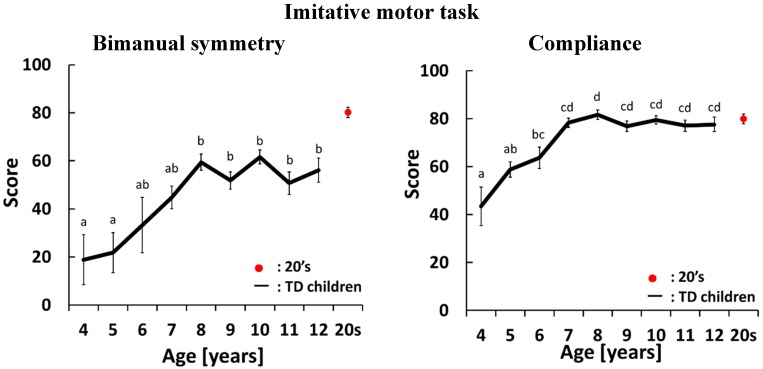

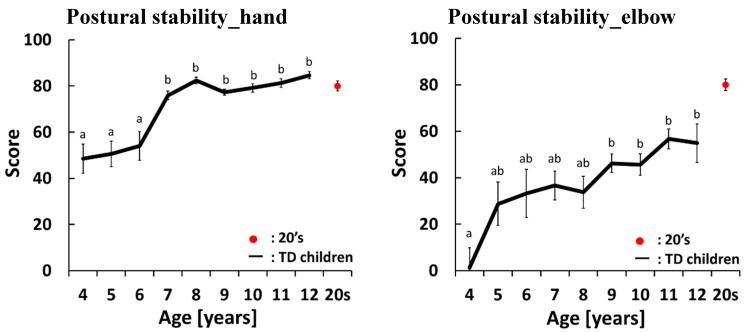
Average and standard error of scores in the imitative motor task. The black line shows the average score for TD children. The red dot shows the average score for participants aged 21–26 years old. There is a significant difference between two different letters which do not include same alphabet on each standard error bars (*p* < 0.05).

**Figure 4:**
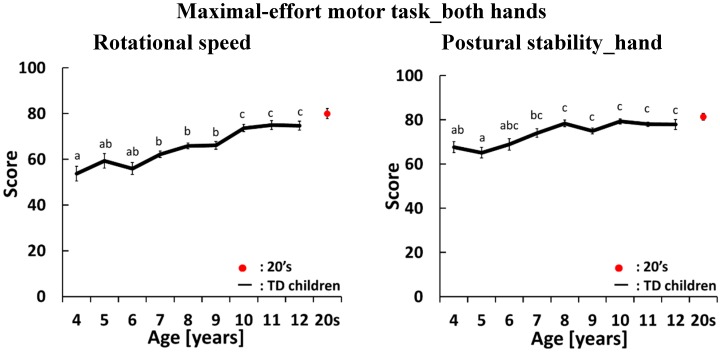

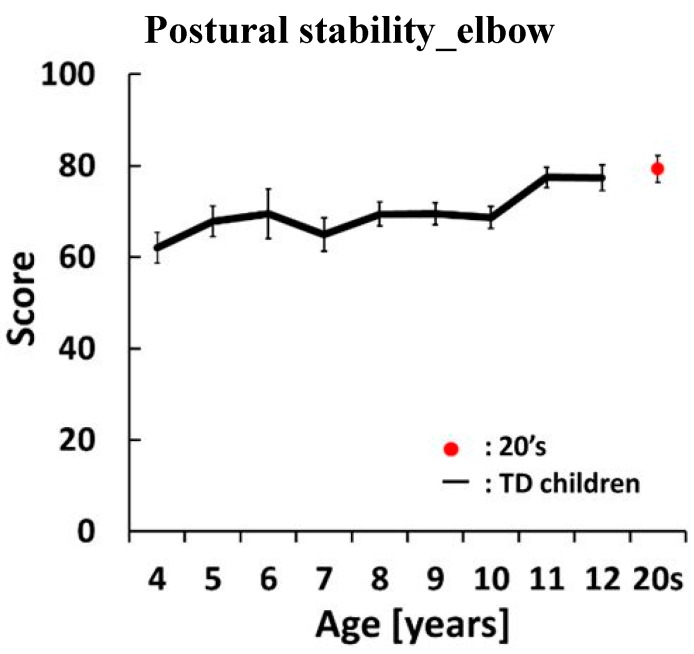
Average and standard error of scores in the maximal-effort motor task performed with both hands. The black line shows the average score for TD children. The red dot shows the average score for participants aged 21–26 years old. There is a significant difference between two different letters which do not include same alphabet on each standard error bars (*p* < 0.05).

**Figure 5:**
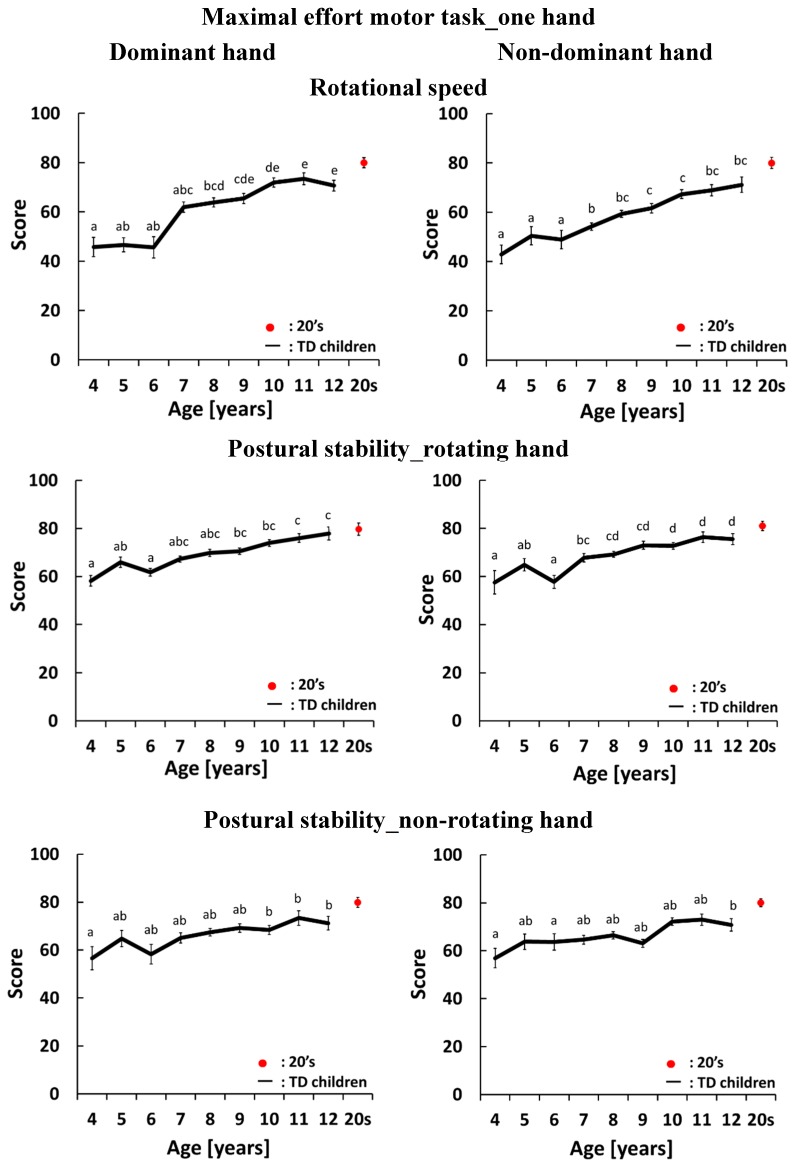
Average and standard error of scores in the maximal-effort motor task performed with one hand. The black line shows the average score for TD children. The red dot shows the average score for participants aged 21–26 years old. There is a significant difference between two different letters which do not include same alphabet on each standard error bars (*p* < 0.05).

**Figure 6:**
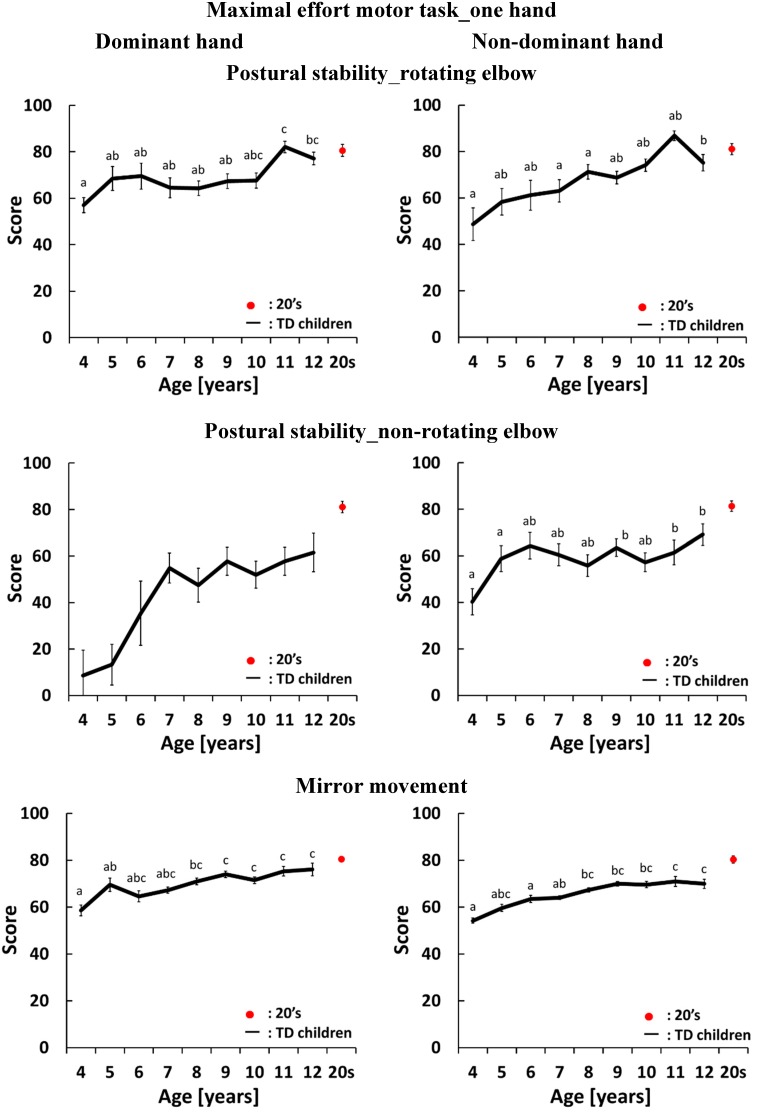
Average and standard error of scores in the maximal-effort motor task performed with one hand. The black line shows the average score for TD children. The red dot shows the average score for participants aged 21–26 years old. There is a significant difference between two different letters which do not include same alphabet on each standard error bars (*p* < 0.05).

Figure 3, Figure 4, Figure 5 and Figure 6 show development curves for pronation and supination quantified using our evaluation system. In these figures, the vertical axis shows the index score and the horizontal axis shows the age of the participants. The black line shows the average score for TD children and the red dot shows the average score for participants aged 21–26 years.

### 4.1. Development Curves for the Imitative Motor Task

In the imitative motor task, we evaluated four indices: bimanual symmetry, compliance with the guide motion, postural stability of the hands and postural stability of the elbows. As shown in Figure 3, all indices had a tendency to increase with age. There were significant differences across age groups for each index. For bimanual symmetry, there were significant differences between 4- and 5-year olds and 8-, 9-, 10-, 11- and 12-year olds. The score increased between 4 and 7 years of age and did not change after 8 years of age. For compliance, there were significant differences between 4-year olds and 7-, 8-, 9-, 10-, 11- and 12-year olds, between 5-year olds and 8-, 9-, 10-, 11- and 12-year olds, and between 6-year olds and 8-year olds. The score increased between 4 and 7 years of age and did not change after 7 years of age. For postural stability of the hands, there were significant differences between 4-, 5- and 6-year olds and 7-, 8-, 9-, 10-, 11- and 12-year olds. The score changed rapidly between 6 and 7 years of age. After 8 years of age the score approached the score achieved by participants aged 21–26 years old and stabilized. For postural stability of the elbows, there were significant differences between 4-year olds and 9-, 10-, 11- and 12-year olds. The score gradually increased with age.

### 4.2. Development Curves for the Maximal-Effort motor Task Performed with Both Hands

In the maximal-effort motor task performed with both hands, we evaluated three indices: rotational speed, postural stability of the hands and postural stability of the elbows, as shown in Figure 4. For rotational speed, there were significant differences between 4-year olds and 8-, 9-, 10-, 11- and 12-year olds, and between 5-, 6-, 7-, 8- and 9-year olds and 10-, 11- and 12-year olds. The score gradually increased with age. For postural stability of the hands, there were significant differences between 4-year olds and 8-, 9-, 10-, 11- and 12-year olds, and between 5-year olds and 7-, 8-, 9-, 10-, 11- and 12-year olds. Score increased slowly between 4 and 8 years of age and did not change after 8 years of age. For postural stability of the elbows, there was no significant difference across ages because variation in this score was large. In this task, all index scores approached the score achieved by participants aged 21–26 years old when children were 12 years old.

### 4.3. Development Curves for the Maximal-Effort Motor Task Performed with One Hand

The development curves for the maximal-effort motor task performed with one hand are shown in Figure 5 and Figure 6. The graphs on the left side of Figure 5 and Figure 6 show the development curves for the task performed with the dominant hand. For rotational speed, there were significant differences between 4-year olds and 8-, 9-, 10-, 11- and 12-year olds, between 5- and 6-year olds and 10-, 11- and 12-year olds, between 7-year olds and 10-, 11- and 12-year olds, and between 8-year olds and 11- and 12-year olds. The score did not change between 4 and 6 years of age, rapidly changed between 6 and 7 years of age, and then gradually increased after 8 years of age. For postural stability of the rotating hand, there were significant differences between 4-year olds and 9-, 10-, 11- and 12-year olds, between 6-year olds and 9-, 10-, 11- and 12-year olds, and between 5-year olds and 11- and 12-year olds. The score gradually increased with age. For postural stability of the non-rotating hand, there were significant differences between 4-year olds and 10-, 11- and 12-year olds. For postural stability of the rotating elbow, there were significant differences between 4-year olds and 11- and 12-year olds, and between 5-, 6-, 7-, 8-, 9- and 10-year olds and 11-year olds. For postural stability of the non-rotating elbow there was a rapid change between 4 and 7 years of age and the score then stabilized between 8 and 12 years of age. However, the variation in this index score was large and there were no significant differences across ages. For mirror movement, there were significant differences between 4-year olds and 8-, 9-, 10-, 11- and 12-year olds, and between 5-year olds and 9-, 10-, 11- and 12-year olds.

The graphs on the right side of Figure 5 and Figure 6 show the development curves for the task performed with the non-dominant hand. For rotational speed, there were significant differences between 4-, 5- and 6-year olds and 7-, 8-, 9-, 10-, 11- and 12-year olds, and between 7-year olds and 10- and 11-year olds. The score increased gradually with age. For postural stability of the rotating hand, there were significant differences between 4-year olds and 7-, 8-, 9-, 10-, 11- and 12-year olds, between 5-year olds and 8-, 9-, 10-, 11- and 12-year olds, between 6-year olds and 7-, 8-, 9-, 10-, 11- and 12-year olds, and between 7-year olds and 10-, 11- and 12-year olds. After 7 years of age, the score increased gradually with age. For postural stability of the non-rotating hand, there were significant differences between 4-year olds, 6-year olds and 11-year olds. For postural stability of the rotating elbow, there were significant differences between 4-, 7- and 8-year olds and 11-year olds. This score increased gradually with age. For postural stability of the non-rotating elbow, there were significant differences between 4- and 5-year olds and 9-, 11- and 12-year olds. For mirror movement, there were significant differences between 4-year olds and 8-, 9-, 10- and 11-year olds, and between 6- and 7-year olds and 11- and 12-year olds.

### 4.4. Radar Charts of Pronation and Supination

**Figure 7:**
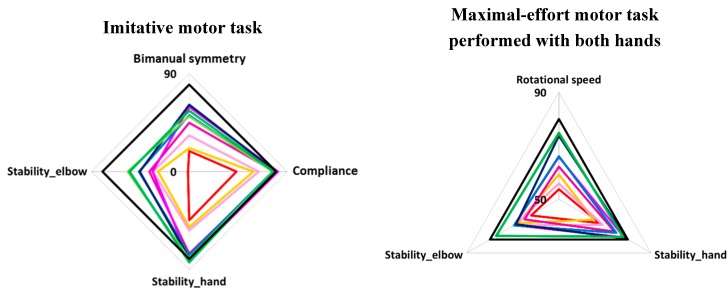

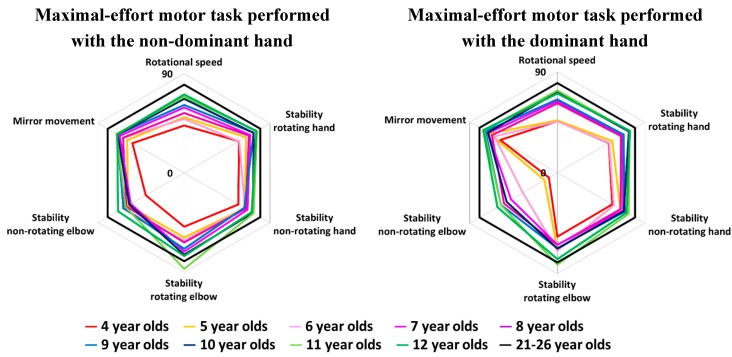
Radar charts for all tasks.

We used a radar chart to represent the balance of these indices (Figure 7). Black lines in Figure 7 show the score of participants aged 21–26 years. The radar charts of children were well-balanced for all ages. From these results, we can observe the developmental changes in pronation and supination that occur with age for each index.

For bimanual symmetry and postural stability of the elbow, the function of children aged 4–6 years was much lower than that of other age groups. After the age of 5 years, function increased, and function stabilized at around 8 years of age. However function did not reach the level seen in participants aged 21–26 years until the age of 12 years. Therefore, it is necessary to measure movement of children aged 13 years and older to confirm the accuracy of our system. We have developed a simple system for quantitatively evaluating age-appropriate pronation and supination. We believe that developmental changes in TD children obtained by our system could become quantitative diagnostic criteria for evaluating developmental disabilities.

Therefore, we plan to compare these developmental changes and scores for developmental disability using this system. We will also investigate the chronological changes in motor function during measurements (10 s) and sex differences in pronation and supination.

## 5. Discussion

There are several SNS examinations, including tests of sensory function, coordination, abnormal or associated movements, repetitive movements, motor speed, accuracy of limb movements, axial movements, balance maintenance and dysrhythmias [2,5,6,8,19]. Pronation and supination are one of these SNS examinations and is used for children aged 4 years and above [13]. In the conventional examination method, children quickly pronate and supinate one hand while the elbows are bent to 90 degrees [4]. Conventionally, the movement is evaluated visually. There are several indices used to evaluate pronation and supination, including excursion of the elbows, dysrhythmias, motor speed and mirror movement [3,4,13,20]. Using SNS examinations, several studies have reported developmental changes in SNS in TD children [2,5].

In this study, we quantified pronation and supination in TD children aged 4–12 years old using a wireless sensor. The indices of pronation and supination that we quantified were bimanual symmetry, compliance, postural stability of the hand, postural stability of the elbow, rotational speed and mirror movements. Bimanual symmetry is an index of coordination between the right and left hands. Compliance is an index of coordination with the movement of the demonstration guide. Postural stability is an index of the excursion of the elbows or hands. Mirror movement is an index of associated movements during the performance of pronation and supination. We found differences in developmental change across the indices.

It was reported that excursion of the elbow decreased between the ages of 4 and 7 years. At 4–5 years of age, excursion was over 15 cm. At 6–7 years of age, excursion was 5–15 cm. Moreover, excursion was less pronounced (under 5 cm) after 8 years of age [13]. In this study, we showed significant differences in postural stability (of the non-rotating elbow) between 4- and 5-year olds and 9-, 11-, and 12-year olds. In the rotating elbow, there was significant difference between 4 year olds and 12 year olds. Moreover, we showed that postural stability of the elbow improved from 8 to 12 years old. Comparison of our result with the results of previous research indicates that we were able to obtain details of developmental change after the age of 8 years compared to conventional criteria.

In our study, rotational speed score increased as age increased, but at 12 years of age it had not reached the level achieved by participants aged 21–26 years. The rotational speed index score did not change between 4 and 6 years of age, rapidly changed between 6 and 7 years of age, and then gradually increased after 8 years of age. Previous studies have reported progressive improvement of motor speed from 5 to 10 years of age [10]. Moreover motor speed is assessed and categorized using the following three grades: late (1–2 Hz), medium (2–3 Hz), and fast (3–4 Hz) [13]. Accordingly, developments in motor speed were observed as the children became older, with those in the late group aged 4–5 years, those in the medium group aged 6–10 years, and those in the fast group aged over 10 years.

There are several studies that have reported developmental changes in mirror movement using visual observation [2,11,21,22,23]. The mirror movements of motor tasks gradually decreased with age [19]. Mirror movements became evident between the ages of 4 and 6 years, decreased between the ages of 7 and 11 years, and were generally not observed after the age of 12 years [13]. However, the largest inter-individual differences were found in children of kindergarten age and in the early school years [2,13]. In our study, there were significant differences in mirror movements between 4-year olds and 8-, 9-, 10-, 11- and 12-year olds, and between 5-year olds and 9-, 10-, 11- and 12-year olds for the dominant hand. For the non-dominant hand, there were significant differences between 4-year olds and 8-, 9-, 10-, 11- and 12-year olds, and between 6- and 7-year olds and 11- and 12-year olds.

In this study, we calculated two novel indices, bimanual symmetry and compliance, to further evaluate pronation and supination. Bimanual symmetry is an index of coordination between the dominant and non-dominant hands. Compliance is an index of coordination with the movement of the demonstration guide. These indices are a novel way of evaluating pronation and supination. Therefore we will discuss the developmental changes of these indices in comparison to previous research of other motor tasks.

Largo and Gasser quantified bimanual coordination and reported developmental changes in bimanual symmetry in children aged 5–18 years [2,7]. Developmental changes in bimanual symmetry during drawing have also been reported between the ages of 4 and 12 years, and the bimanual symmetry of 4- and 5-year olds was inferior to that of children aged 6 years and older [24]. In our study, bimanual symmetry improved between the ages of 4 and 8 years and stabilized between the ages of 8 and 12 years. And we found a significant difference in bimanual symmetry between 4- and 5-year olds and 8-, 9-, 10-, 11- and 12-year olds. Our results are consistent with previous results reported in other developmental studies and show more detailed developmental change after the age of 6 years.

In our study, compliance improved between the ages of 4 and 7 years and stabilized after the age of 7 years. The level of performance of 12-year olds reached that of participants aged 21–26 years. In a follow-a-finger test in which compliance was quantified by visual observation, performance of 4- and 5-year olds was especially low in comparison to performance of children aged 6 years and older [13]. These results are consistent with results reported in other developmental studies.

## 6. Conclusions

In this study, we quantified developmental changes in pronation and supination as a first step. Results obtained using our system showed developmental changes that were consistent with criteria for SNS. Moreover we were able to obtain more details on developmental change than have been obtained in several previous research studies that have used visual observation. Our results indicate that it may be possible to use our system as quantitative evaluation method for developmental disorder. It has not been fully verified whether our proposed development curves were more useful and quantifiable compared to visual observation or not. In this manuscript, we focused on the quantifying development curve of pronation and supination which is one of SNS examination using data of TD children. Before our experiment, we checked age, sex, birthday, and dominant hands in TD children. We have not evaluated TD children by visual observation or rating scale for developmental disorders. Therefore, we will compare our proposed development curves with data of children with developmental disorders or conventional methods in future work.
